# The Role of Blinks, Microsaccades and their Retinal Consequences in Bistable Motion Perception

**DOI:** 10.3389/fpsyg.2021.647256

**Published:** 2021-04-08

**Authors:** Mareike Brych, Supriya Murali, Barbara Händel

**Affiliations:** Department of Psychology III, University of Würzburg, Würzburg, Germany

**Keywords:** eye movements, spontaneous eye blink, microsaccade rate, microsaccade direction, bistable perception, ambiguous plaid 4

## Abstract

Eye-related movements such as blinks and microsaccades are modulated during bistable perceptual tasks. However, if they play an active role during internal perceptual switches is not known. We conducted two experiments involving an ambiguous plaid stimulus, wherein participants were asked to continuously report their percept, which could consist of either unidirectional coherent or bidirectional component movement. Our main results show that blinks and microsaccades did not facilitate perceptual switches. On the contrary, a reduction in eye movements preceded the perceptual switch. Blanks, on the other hand, thought to mimic the retinal consequences of a blink, consistently led to a switch. Through the timing of the blank-introduced perceptual change, we were able to estimate the delay between the internal switch and the response. This delay further allowed us to evaluate that the reduction in blink probability co-occurred with the internal perceptual switch. Additionally, our results indicate that distinct internal processes underlie the switch to coherent vs. component percept. Blanks exclusively facilitated a switch to the coherent percept, and only the switch to coherent percept was followed by an increase in blink rate. In a second study, we largely replicated the findings and included a microsaccade analysis. Microsaccades only showed a weak relation with perceptual switches, but their direction was correlated with the perceived motion direction. Nevertheless, our data suggests an interaction between microsaccades and blinks by showing that microsaccades were differently modulated around blinks compared with blanks. This study shows that a reduction in eye movements precedes internal perceptual switches indicating that the rate of blinks can set the stage for a reinterpretation of sensory input. While a perceptual switch based on changed sensory input usually leads to an increase in blink rate, such an increase was only present after the perceptual switch to coherent motion but absent after the switch to component percept. This provides evidence of different underlying mechanism or internal consequence of the two perceptual switches and suggests that blinks can uncover differences in internal percept-related processes that are not evident from the percept itself.

## Introduction

Spontaneous eye blinks are strongly influenced by sensory input (Ohdra, 1995; Siegle et al., 2008; Oh et al., 2012; Bonneh et al., 2016; Hoppe et al., 2018). Additionally, they are closely linked to cognitive processes that influence the perceptual outcome (Ito et al., 2003; van Dam and van Ee, 2005; Nakatani et al., 2011; Otero-Millan et al., 2012; Grossman et al., 2019; Ang and Maus, 2020; Brych and Händel, 2020; Maus et al., 2020). Similarly, microsaccades are related to perceptual (Martinez-Conde et al., 2006; Rolfs et al., 2008; Ko et al., 2010; McFarland et al., 2015; Intoy and Rucci, 2020) and attentional processes (Laubrock et al., 2005; Valsecchi et al., 2007; Pastukhov and Braun, 2010; Hicheur et al., 2013; Pastukhov et al., 2013; Gao et al., 2015). The relationship between these eye movements and internal perceptual processes is still not clear. One way to study this is by looking at ambiguous stimuli, wherein a single unchanging stimulus can be interpreted in two or more ways (Necker, 1832; Rubin, 1921; Wallach, 1935; Adelson and Movshon, 1982). In such stimuli, any change in perception reflects an internal process and not an external change.

There have been attempts to understand the role of eye movements during internal perceptual switches using such ambiguous stimuli. Conducting a time-resolved analysis, some studies have found a reduction in microsaccade rate before and an increase after the perceptual switch report, using the apparent motion stimulus (Laubrock et al., 2008) and the slant-rivalry stimulus (van Dam and van Ee, 2006). According to these authors, this modulation was likely a consequence of the switch (van Dam and van Ee, 2006). Pertaining to the drop in microsaccade rate, Laubrock et al. (2008) discuss the possibility that the modulation could result from the switch itself and could be associated with the perceptual decision (Laubrock et al., 2008). The same authors (Laubrock et al., 2008) show that microsaccade directions causes a bias in the perceived motion direction. Others have also suggested such a causal role of microsaccades. For instance, Otero-Millan et al. (2012) reported that microsaccades increased before perceptual switches in the rotating snake illusion. Similarly, Troncoso et al. (2008) found that higher microsaccade rates were associated with faster motion perception while viewing the Enigma illusion.

Concerning blinks, only a few studies have analyzed blink modulation around ambiguous perceptual switches in a time-resolved manner. Ito et al. (2003) for instance, using a version of the triangles of Attneave (1968), found that blinks decreased before and increased after the perceptual switch report. A similar finding was reported in another study (van Dam and van Ee, 2005, 2006), using the slant-rivalry stimulus.

Our goal was to understand if eye movements are a cause or consequence of these transitions. On the one hand, these perceptual transitions could alter the ongoing eye movement rate, for example, due to attention diverted toward them. On the other hand, eye movements could also trigger switches. This effect could, of course, be due to the visual disruptions that accompany them. Therefore, to dissociate the effect of eye movements from their visual consequences, we added external disruptions to the stimulus that mimic the said eye movements. In experiment 1, we tested the role of blinks and dissociated influences mediated by their retinal consequences by adding short interruptions of the visual input. In experiment 2, we additionally analyzed the causal or consequential role of microsaccades and added small shifts.

Additionally, in both experiments, we accounted for the possible influence of the movement of eye ball during blinks and microsaccades by testing four different stimulus versions. For instance, during blinks, it has been reported that the eyeball moves mainly downward (Collewijn et al., 1985), whereas microsaccades during fixation are mainly executed in the horizontal direction. As these eye movements might preferably lead to a switch toward or away from the executed movement direction, we tested four different stimulus rotations resulting in either the coherent or the component motion moving in a cardinal direction (see Methods section).

We used the ambiguous plaid stimulus, which consists of moving gratings superimposed over each other (von Schiller, 1933; Wallach, 1935; Adelson and Movshon, 1982; Hupé and Rubin, 2004). The stimulus is seen either as one single grating (coherent percept) or as two separate gratings (component percept). The ambiguity arises due to the aperture problem (Binder et al., 2009).

Our main results showed that blinks and microsaccades did not facilitate perceptual switches. On the contrary, a reduction in eye-related movements preceded the perceptual switch. Blanks, on the other hand, thought to mimic the retinal consequences of a blink, consistently facilitated a switch in percept. This allowed us to mark the time period in which the perceptual switch likely occurred, indicating that the reduction in blink probability was not a result of the perceptual switch but temporally co-occurring. We additionally found that blinks succeeding the switch were modulated in a percept-specific manner showing a significant increase only for one type of perceptual switch. This deviation in blink behavior suggests a difference in the internal process associated with the two perceptual switches. Such an additional difference is not evident from the subjective perceptual experience.

## Materials and Methods

The study consisted of two experiments using a very similar stimulus and setup. Experiment 2 was conducted as a replication study and additionally used an eye tracker that allowed us to analyze microsaccades in addition to blinks.

### Stimulus

**Figure 1:**
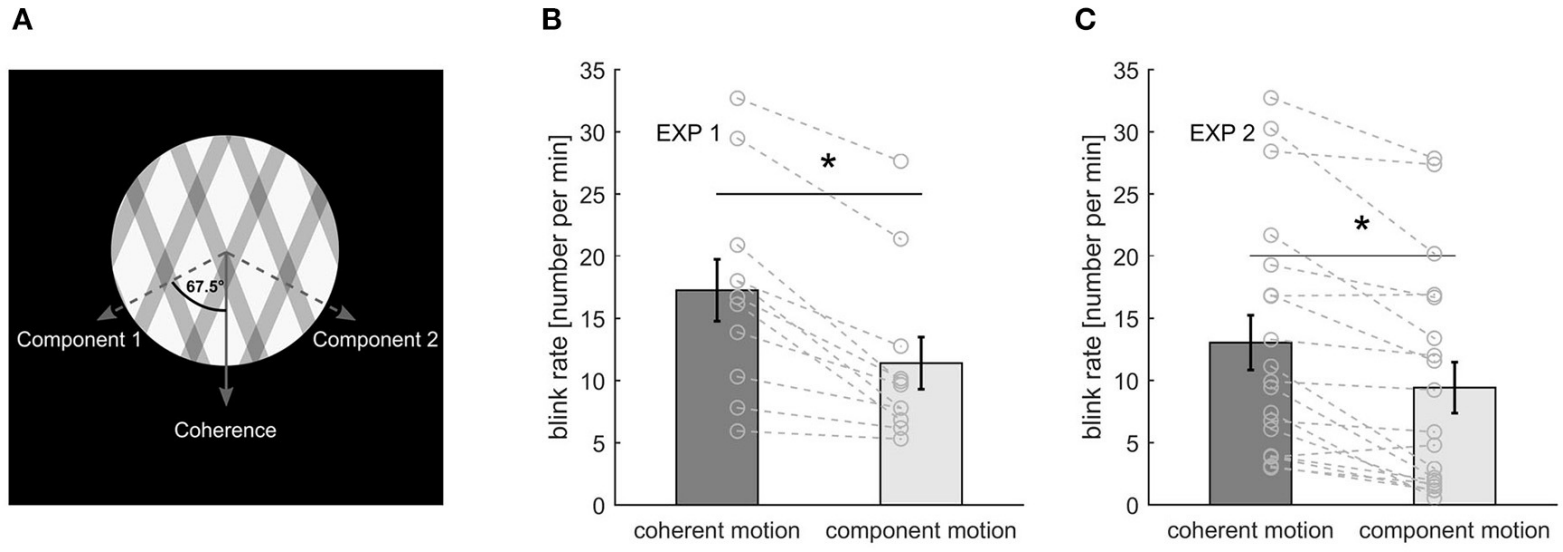
**(A)** Stimulus representation. **(B)** Blink rate during the different percepts in experiment 1. **(C)** Blink rate for the different percepts in experiment. Bars and error bars represent mean ± standard error of the mean (SEM). Gray lines represent data of individual participants. An asterisk marks a significant difference at *p* < 0.001.

A moving grating displayed behind a fixed-size aperture is usually perceived as moving perpendicular to the parallel lines of the grating. Superimposing another grating with a different orientation creates the ambiguous plaid stimulus: The two gratings can either be perceived on top of each other as two components with different directions or as a unified plaid pattern coherently moving in one direction. For the stimulus with 0° rotation, the coherent plaid pattern moved directly downward, and the components moved in angles of ±67.5° to the coherent motion direction (Figure 1A). The lines of the grating were square-waved with a width of 0.5° and a spacing of 1.8° in experiment 1, and a width of 0.4° and a spacing of 1.4° in experiment 2. Line color was 180 on an 8-bit grayscale (0-black, 256-white), while the intercept color was 120. The overall size, speed, and rotation of the moving grating is specified for each experiment, separately (please see below).

The experimental program was implemented in MATLAB, using the Psychophysics Toolbox extensions (Brainard, 1997; Pelli, 1997; Kleiner et al., 2007). Response buttons were stuck on the table being connected to a BBTK response box (model: K-RB1-4; The Black Box ToolKit Ltd, UK), which was connected to a laptop via USB. Participants indicated their prevalent percept by continuously pressing one of two buttons with their right index finger only. Lifting the button was the first indicator that the percept changed, which is why we present our results in relation to the button lift instead of the later-happening button press.

### Experiment 1

#### Participants

Fourteen psychology students of the University of Würzburg [age: 20.5 ± 2.18 years (mean ± SD)] took part in the first experiment. They received study credit for their participation. All participants had normal or corrected-to-normal vision. The study was approved by the local ethics committee and complied with the European data protection law (DSGVO). The participants gave their written informed consent before taking part in the study.

#### Procedure

Participants were seated in a dark room 40 cm away from the screen with their heads kept in a fixed position using a chin rest. For stimulus presentation, we used a NEC MultiSync monitor (1,280 × 1,024 resolution, 60 Hz refresh rate), which was controlled by a Dell Precision (M6700) laptop running Windows 10. Binocular eye movements were recorded with 120 Hz using SMI eye tracking glasses (SensoMotoric Instruments GmbH, Berlin, Germany). Although we mentioned that eye gaze as well as blinks can be recorded with the eyetracker, participants were naïve to our intention to analyze blinks.

The stimulus was presented in an aperture of 7.2° in diameter, on a black background, and the gratings moved with a speed of 0.9°/s. A red fixation spot of 0.3° in diameter was placed in the center of the stimulus (Figure 1A).

During a spontaneous eyeblink, the eyeball has been reported to slightly move downward and inward (Collewijn et al., 1985). To test for a possible influence of the vertical movement of the eyeball during blinks on the percept, four different stimulus rotations were presented: 0°, 67.5°, 112.5°, and 180°, with respect to the coherent motion. These specific rotations were chosen such that either the coherent motion of the gratings moved up (180°) or downward (0°), or one of the gratings (components) moved up (112.5°) or downward (67.5°).

To control for the visual changes during a blink, the screen was blackened for a random duration between 116 and 167 ms randomly every 3 to 6 s (in steps of 0.5 s) in half of the trials, which is similar to blink characteristics and will be referred to as “blanks.”

The first experiment consisted of eight trials with a duration of 6 min each. Each of the four stimulus rotations were presented twice, once with blanks, once without. Trial order was completely randomized. A one-point calibration of the SMI eyetracker was performed prior to the start of the experiment.

### Experiment 2

#### Participants

Twenty-two new participants [15 females, age: 27.4 ± 8.88 years (mean ± SD)] took part in the second experiment. They received payment or study credit for their participation. All participants had normal or corrected-to-normal vision. They gave their written informed consent prior to the participation. The study was approved by the local ethics committee and complied with the European data protection law (DSGVO).

#### Procedure

Participants were seated in a darkened room and placed their head on a chin rest 68 cm away from the monitor. The stimulus was presented on a Mitsubishi Diamond Pro 2070SB monitor (1,152 x 864 resolution, 60-Hz refresh rate). The experiment was controlled by a Tuxedo laptop running Ubuntu 16.04 LTS. To analyze very small eye movements, binocular eye movements were recorded at 500 Hz using an EyeLink 1000 eyetracker (SR Research, Ottawa, Ontario, Canada). Similar to experiment 1, we mentioned that the eyetracker is able to record various eye movements [blinks, (micro-)saccades, drift, …], but participants were naïve to our explicit analysis of blinks and microsaccades.

The stimulus had a diameter of 5.8°, and the coherent pattern moved with a speed of 0.7°/s. The fixation spot was 0.25° in diameter.

Again, the influence of the vertical movement of the eyeball during a blink and the horizontal movement of the eyeball during a microsaccade were controlled with four stimulus rotations. We used again the stimulus rotations of 0° and 67.5° (blink related) and added rotations of 22.5° (coherent motion, i.e., one component moving horizontally to the left, the other one to the bottom right) and 90° (coherent motion horizontally to the left) (microsaccade related). Our Supplementary Material include illustrations of the rotations.

In addition to the blanking trials, microshift trials were introduced to control for the visual changes during a microsaccade. During microshift trials, the stimulus randomly shifted every 3 to 6 s randomly toward the right or the left by 0.2°. While the size of the microshift was similar to a real microsaccade, we reduced the rate for two reasons. First, microshifts are clearly visible to the observer, and if they are presented as often as microsaccades, they introduce a sort of jitter. This might interfere with the resulting percept possibly forming intermediate or additional perceptual interpretations. Second, to have a within-condition control, we preferred to have periods with and without microshifts for data analysis. The fixation spot stayed at its position. The maximal deviation of the stimulus from the original position was 0.8°, i.e., the shift could maximally happen four times in the same direction.

The second experiment consisted of two blocks each having eight trials of 3-min duration in random order. Before each block, a five-point calibration and validation of the Eyelink eye tracker was performed. Each block consisted of four test trials (all four rotations described above), two blank trials to simulate blinks (rotations 0° and 67.5°) and two microshift trials to simulate microsaccades (rotations 22.5° and 90°).

### Data Analyses (Experiments 1 and 2)

For the first experiment, two participants were excluded because of a lower blink rate than 5 blinks/min, another one due to more than 39% of missing data. For the second experiment, one participant was excluded due to very high blink rate (>35 blinks/min) and two more due to a blink rate lower than 5 blinks/min during all trials without blanks or microshifts.

The identical trials of block one and two in the second experiment were concatenated before analysis. Event (blinks, blanks, microsaccades, and microshifts) onsets were counted for bins every 100 ms around the button lifts indicating perceptual switches. If such an event was detected around multiple switches, we divided the counts by the number of occurrences. To incorporate different switch rates, we averaged the time course over all switches. Furthermore, we controlled for different rates of eye movements by dividing the result by the number of eye movements during the trial. Finally, these time series were z-transformed and averaged over trials. These time series around a switch were compared with time series where no switch occurred, i.e., all possible non-overlapping 4-s time periods without a perceptual change starting at 2 s after the last perceptual switch. Every second of these no-switch periods was used for comparison with a switch to coherent motion and every other no switch period for comparison with a switch to component motion.

Cohen's d for paired sample *t*-tests was calculated as the mean of D divided by the standard deviation of D, where D is the differences of the paired sample values.

For statistical analysis, we implemented the non-parametric statistical test described by Maris and Oostenveld (2007), which is based on clustering of adjacent time samples that show a similar difference in sign and magnitude. The threshold for clustering was selected as the 97.5 quantile of a T-distribution. Critical *t*-values were approximated by a Monte Carlo estimate, which was calculated on 1,000 random partitions and a critical alpha-level of 0.05.

### Blink Detection (Experiments 1 and 2)

Whenever the eyelid covers the eye, rapid changes in pupil diameter are recorded by the eyetracker. Therefore, we developed a blink detection algorithm based on pupil diameter. First, pupil diameter was z-transformed. By visual inspection, a manually set amount of standard deviations (between 1.9 and 4) of the z-transformed pupil diameter was chosen for threshold. A blink was assumed when z-transformed pupil diameters of both eyes decreased below this threshold (pupil was partly covered by the eye lid during the start/end of a blink) or if the pupil was not detected at all (pupil was fully covered by the eye lid). The start and the end of the blink were then extended until the pupil diameter of both eyes were higher than half the threshold. Blinks less than 100 ms apart from one another were concatenated, and blinks shorter than 50 ms or longer than 1,000 ms were discarded.

### Microsaccade Detection (Experiment 2)

We implemented an algorithm based on the description by Engbert and Kliegl (2003) where a transformation of fixation positions to two-dimensional velocity space is performed to detect (micro-)saccades using their high peak velocities. We assumed a minimal duration of four samples (8 ms) and only considered binocular (micro-)saccades. In line with previous research, (micro-)saccades showed a linear relation of amplitude and peak velocity known as “main sequence” (Zuber et al., 1965). Microsaccades were defined by an amplitude of maximally 1°. Furthermore, we excluded microsaccades based on a velocity criterion (0.2% of all microsaccades) as well as around blinks (0.01% additional excluded). For more details, please refer to our Supplementary Material.

## Results

During the first experiment, participants perceived coherent motion for a longer total amount of time than component motion [21.03 ± 21.83 s compared with 8.06 ± 8.03 s (mean ± standard deviation); paired *t*-test: *t*_(10)_ = 2.16, *p* = 0.056, *d* = 0.65]. Coherent motion was also dominant in the second experiment [*t*_(18)_ = 4.61, *p* < 0.001, *d* = 1.06], but percepts switched faster [percept durations of 14.77 ± 6.65 s compared with 7.57 ± 4.41 s (mean ± SD) for coherent and component motion, respectively]. The duration of percept was calculated between a button press and the corresponding lift, and revealed the typical unimodal and positively skewed distribution when plotted as histograms (not shown).

### Blinks (Experiments 1 and 2)

During the first experiment, participants blink on average 11.12 ± 5.39 (SD) times per minute with a mean duration of 136.80 ± 26.80 ms (SD). For the second experiment, the blink rate was 12.09 ± 8.83 (SD) blinks per minute with a mean blink duration of 171.32 ± 46.47 ms (SD). Furthermore, we calculated the blink rate separately for the different percepts taking into account the respective percept durations. During both experiments, participants blinked significantly more during coherent motion than during component motion [experiment1: *t*_(10)_ = 5.56, *p* < 0.001, *d* = 1.68; experiment 2: *t*_(18)_ = 4.86, *p* < 0.001, *d* = 1.12] (Figures 1B,C). Blanks were slightly shorter than blinks in both experiments with a mean length of 125 ± 19 ms (SD) and 141 ± 19 ms (SD), respectively.

To investigate if a change in perception is linked to a blink event, we looked at the normalized blink rate around perceptual switches, separately for switches to coherent and component motion and statistically compared it with the normalized blink rate when no switch occurred. The same was done for the normalized blank rate. This was done to assess if any influence was introduced by the visual consequences of the eye closure during a blink, as mimicked by the blank. Normalized blink rates were taken from trials without blanks or microshifts, but were combined over stimulus rotations. Please note that no *p*-values are reported due to the non-parametrical statistical testing that was applied (Maris and Oostenveld, 2007).

**Figure 2:**
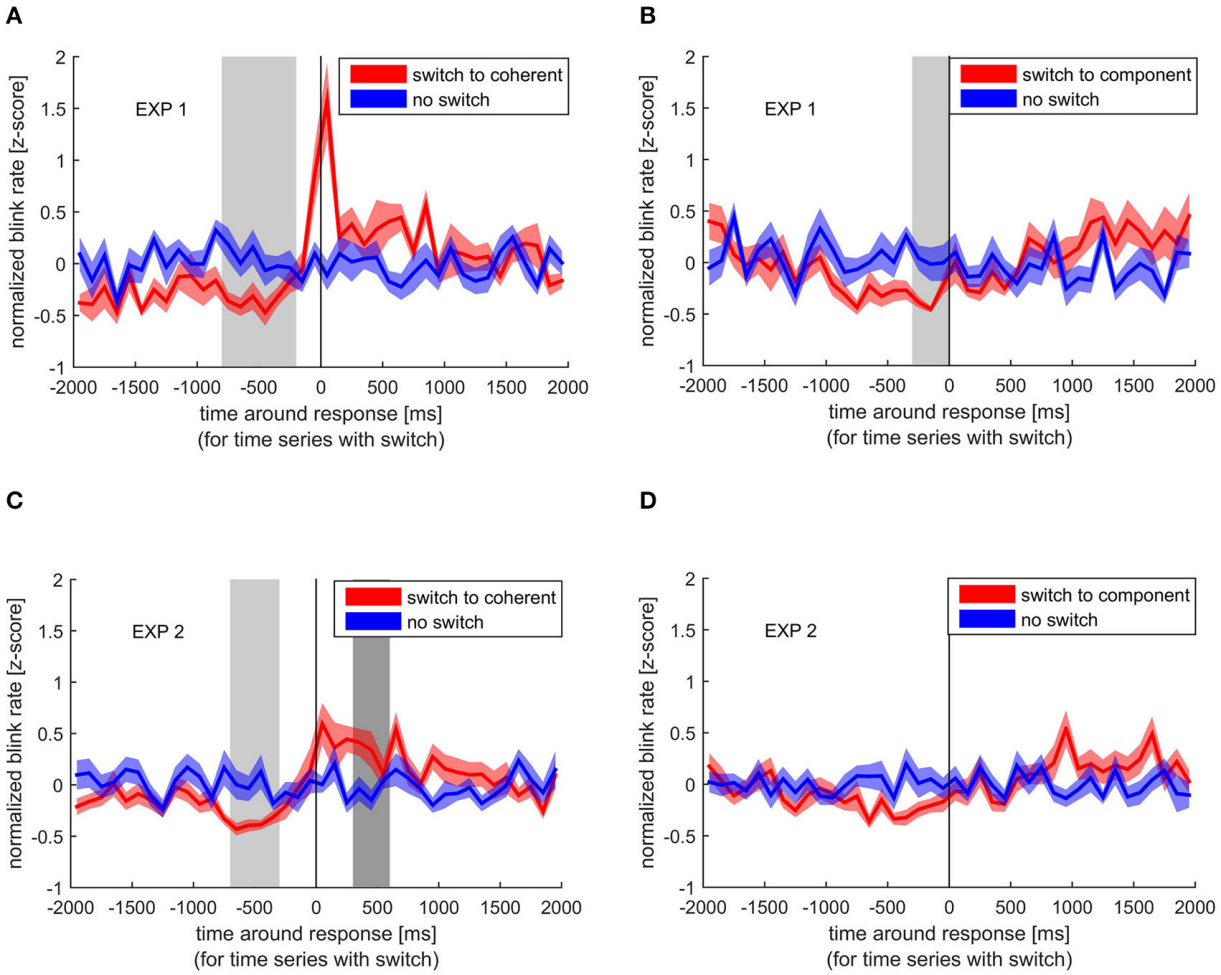
Normalized blink rate around the response indicating a perceptual switch (red) compared with the normalized blink rate during no switch (blue). Colored lines and ribbons represent mean ± standard error of the mean (SEM). Vertical shaded areas mark significant time points revealed by the non-parametrical statistical test procedure described by Maris and Oostenveld (2007). **(A)** Switch to coherent motion in experiment 1. **(B)** Switch to component motion in experiment 1. **(C)** Switch to coherent motion in experiment 2. **(D)** Switch to component motion in experiment 2.

When switching to coherent motion, there was a significant decrease in blink rate between −800 and −200 ms before the button lift (indicating a perceptual switch) in the first experiment compared with time periods with no switch. This decrease was replicated in the second experiment, where we found significant differences between −700 and −400 ms (Figure 2). When switching to component motion, such a decrease in blink rate was found in the first experiment (−300 to 0 ms before the switch), but did not reach significance in the second experiment, although a decrease before the switch is clearly visible. Interestingly, blink rate strongly increased around the time of a response indicating a switch to coherent motion. This peak in blink rate is clearly visible in both experiments, but statistical comparison between blink rate around a switch and around no switch only reached significance in the second experiment between 300 and 600 ms after the response.

**Figure 3:**
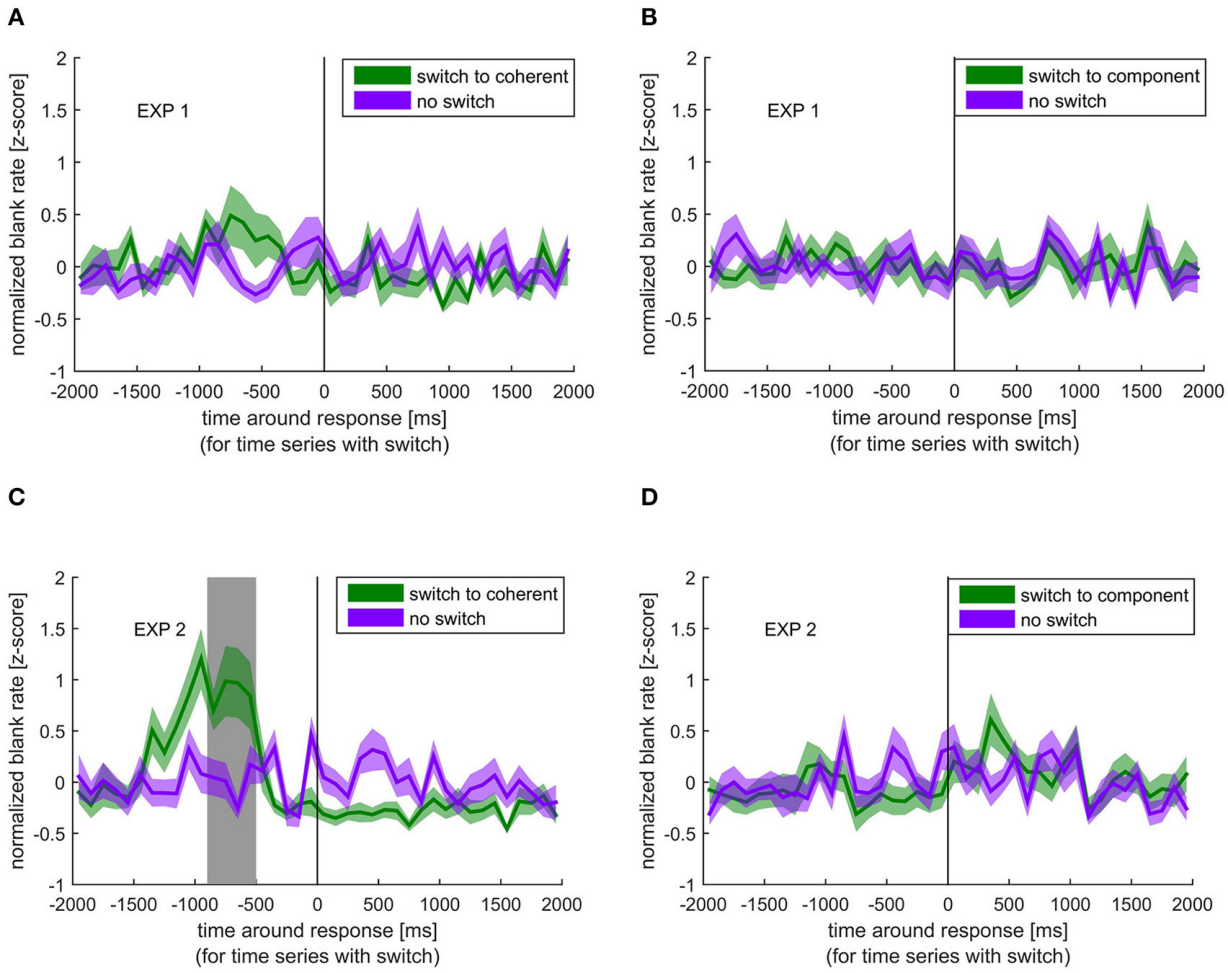
Normalized blank rate around the response indicating a perceptual switch (green) compared with the normalized blank rate during no switch (purple). Colored lines and ribbons represent mean ± standard error of the mean (SEM). Vertical shaded area marks significant time points revealed by the non-parametrical statistical test procedure described by Maris and Oostenveld (2007). **(A)** Switch to coherent motion in experiment 1. **(B)** Switch to component motion in experiment 1. **(C)** Switch to coherent motion in experiment 2. **(D)** Switch to component motion in experiment 2.

In contrast to the blink rate modulation, blanks, although again showing the strongest effect for switches to coherent motion, showed a different temporal pattern. As shown in Figure 3, the blank rate increased before the switch to coherent motion in experiment 1, which was even more pronounced in experiment 2. This increase in blank rate around the switch to coherent motion was significantly different from the blank rate around no switch between −900 and −500 ms before the response in the second experiment. This pattern was not visible when switching to component motion. When looking at the different stimulus rotations separately, all patterns were very similar, which means that the effect of blanks and blinks are independent of the movement direction of the stimulus (see Supplementary Material).

### Microsaccades (Experiment 2)

Due to the low sampling frequency of the eyetracker used in experiment 1, we were only able to analyze microsaccades in the second experiment.

Over all trials and participants, we found a microsaccade rate of 1.39 ± 0.40/s (mean ± SD). Looking at the different percepts, a paired *t*-test revealed that participants had a significantly higher microsaccade rate during coherent motion (1.42 ± 0.40/s) than during component motion [1.29 ± 0.38/s; *t*_(18)_ = 2.43, *p* = 0.026, *d* = 0.56] taking into account the respective percept durations. Coherent percept is therefore associated with a higher microsaccade rate as well as with a higher blink rate compared with the component percept.

**Figure 4:**
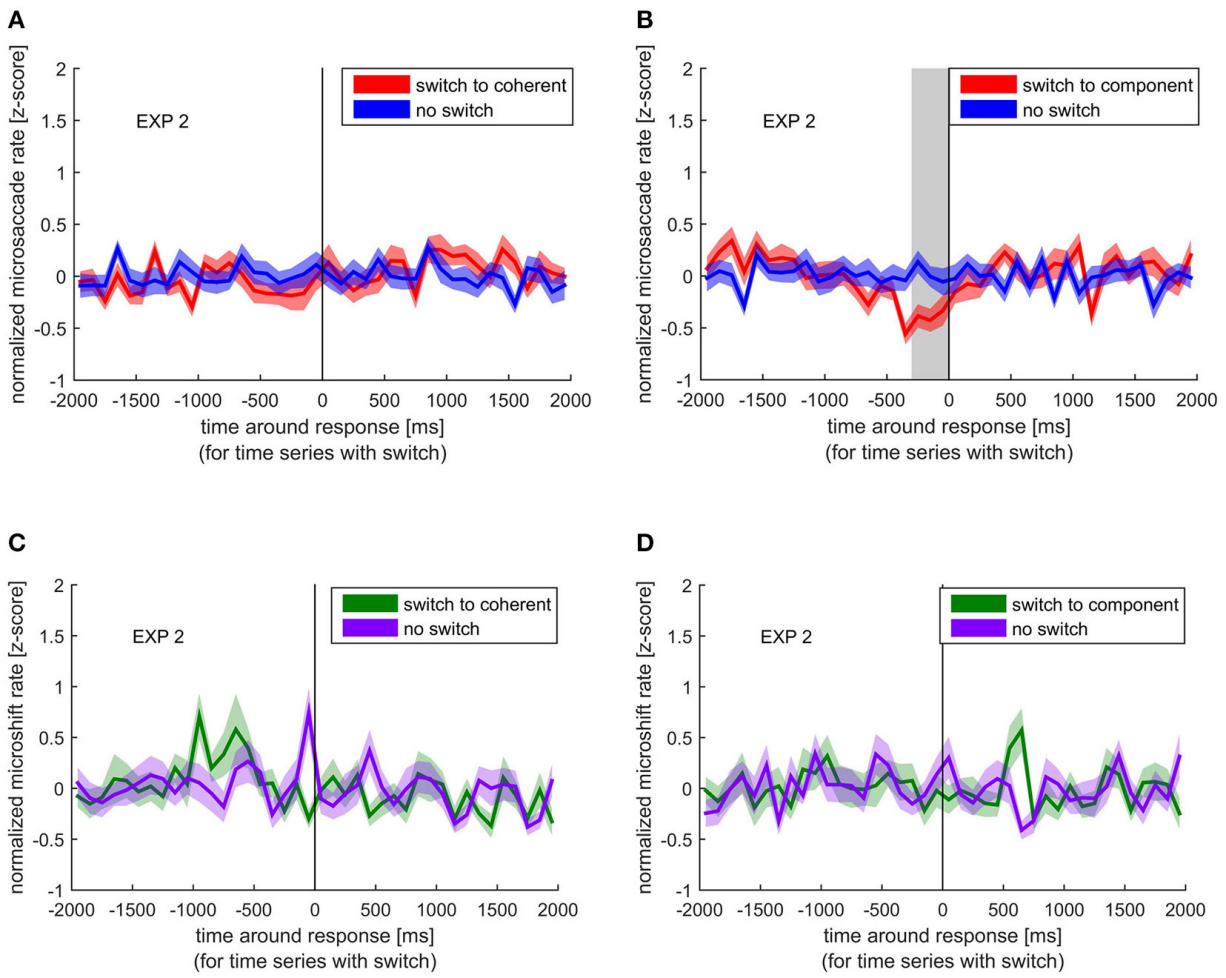
Normalized microsaccade/microshift rate around the response indicating a perceptual switch (red/green) compared with the normalized microsaccade/microshift rate during no switch (blue/ purple). Colored lines and ribbons represent mean ± SEM. Vertical shaded area marks significant time points revealed by the non-parametrical statistical test procedure described by Maris and Oostenveld (2007). **(A)** Microsaccade rate around switch to coherent motion. **(B)** Microsaccade rate around switch to component motion. **(C)** Microshift rate around switch to coherent motion. **(D)** Microshift rate around switch to component motion.

Similar to the comparison of normalized blink rate around perceptual switches and no switches, we looked at the differences between normalized microsaccade rate around switches and no switches. To assess the specific influence of the visual shift accompanied by a microsaccade (as mimicked by the microshift), we compared microshift rate around switches and no switches (Figure 4).

Similar to the blink rate decrease before a switch, we found a significant decrease in microsaccade rate between −300 and 0 ms before the switch to component motion. However, such a decrease was not visible before a switch to coherent motion. Microshifts showed a different pattern, which resembles the blank rate pattern with respect to the increase before a switch to coherent motion, but the difference between normalized microshift rate before or after any switch compared with no switch was not significant.

**Figure 5:**
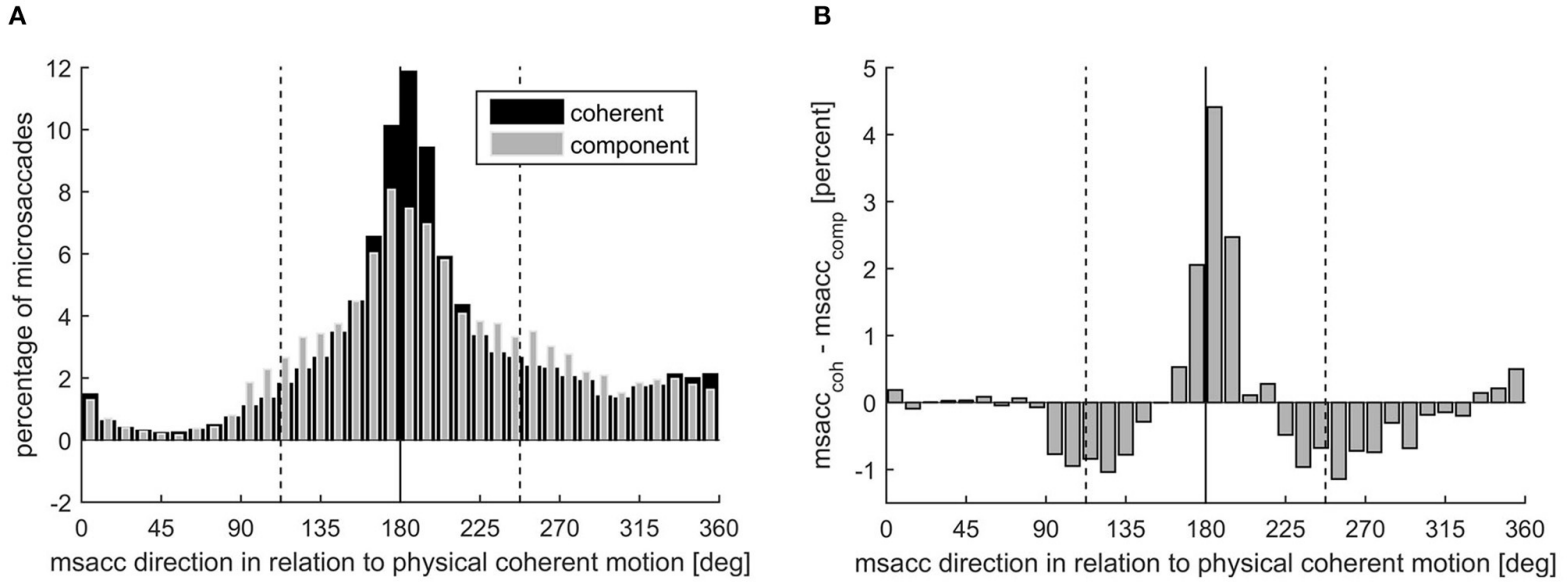
**(A)** Microsaccade direction presented as microsaccade number in percent during coherent (black) and component motion (gray) perception, separately. The solid line marks the opposite direction of the coherent motion, while the dashed lines mark the opposite directions of the component motions. **(B)** Microsaccade direction presented as the difference in microsaccade number during coherent motion and component motion perception in percent.

In addition to the analysis of microsaccade rate, we explored the direction of fast eye movements. During the perception of the stimulus, fast eye movements with typical microsaccadic characteristics could be observed in the direction opposite to the stimulus motion. Independent of the percept, we found that the main direction of microsaccades was opposite to the coherent motion direction. After calculating the percentages of microsaccades for all directions in steps of 10° (Figure 5), we found 21.99% of all microsaccades during coherent percept directed opposite to the physical movement of the coherent motion (180° ± 10°), but also 15.53% during component percept share this direction.

Despite this clear dominance of the direction opposite to the coherent motion, there was an influence of the percept on the distribution of the microsaccade direction circumstantiated by the significantly higher percentage of microsaccades in this direction during coherent percept (mean ± SD: 21.99 ± 9.48%) than during component percept [15.53 ± 4.32%; *t*_(18)_ = 3.57, *p* = 0.002, *d* = 0.82].

Accordingly, significantly more microsaccades were directed opposite to the component motions (112.5° ± 10° and 247.5° ± 10°) when component motion was perceived (12.70 ± 2.25%) compared with when coherent motion was perceived [8.63 ± 2.93%; *t*_(18)_ = −5.84, *p* < 0.001, *d* = 1.34].

### Relationship Between Eye Blinks and Microsaccades

**Figure 6:**
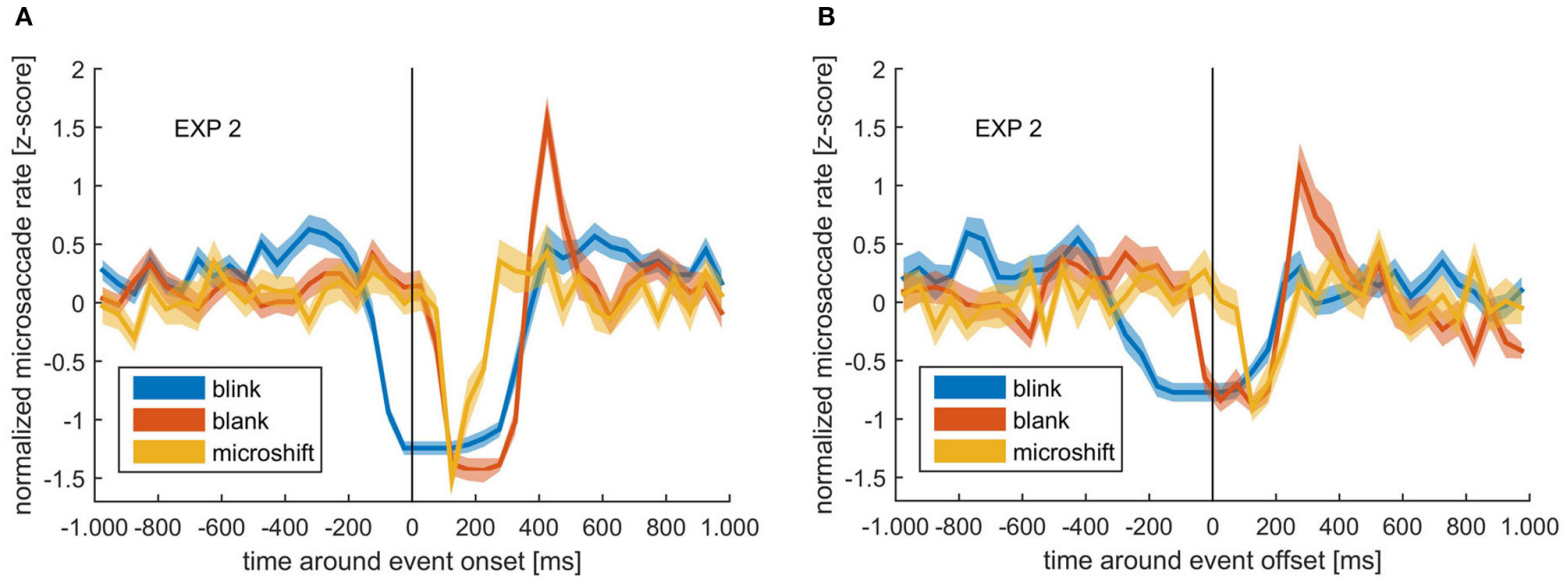
**(A)** Normalized microsaccade rate around blink *onset* (blue), blank *onset* (red), and microshift (yellow). **(B)** Normalized microsaccade rate around blink *offset* (blue), blank *offset* (red), and microshift (yellow). Colored lines and ribbons represent mean ± SEM.

A normalized microsaccade rate (within 50-ms bins) around external sensory changes (blanks and microshifts) and internally introduced sensory changes (blinks) is depicted in Figure 6 aligned to either blink or blank on- or offset. Microshifts consisted of a change between two frames, so the onset is equal to the offset. A pronounced reduction in microsaccade rate could be observed around all events. However, the microsaccade rate decrease started at different time points for external events (blank and microshift) compared with the internally introduced blinks. While the rate dropped immediately after the onset of the (unpredictable) blanks and microshifts, the decrease started already 200 ms before a blink. The quick closure and opening of the eye during a blink can lead to the wrong detection of saccadic events. However, due to the observed long alteration in microsaccade rate around blinks, our conservative exclusion of microsaccades 20 ms around a blink (see Methods section) is unlikely to have influenced this outcome.

Looking at the time after the onset of events, there was a clear peak in microsaccade rate at ~400 ms after blank onset, but not for microshifts or blinks. Note that the reduction before blink onset is a real rate modulation, while the low rate after blink onset is due to the fact that the eye is closed, and therefore, no microsaccades can be detected using a video-based eye tracker.

## Discussion

We examined the time-resolved rate of eye blinks and microsaccades during perceptual bistability of the ambiguous plaid stimulus. We found that eye blinks decrease before and increase after the reported perceptual switch depending on the percept. When examining the two types of perceptual switches (coherent vs. component motion) separately, there was a difference in modulation, with only the switch to coherent motion being accompanied by an increase after the perceptual switch report. Microsaccades also showed a percept-specific modulation in their rate, with a decrease specifically before the report of the switch to the component percept. Additionally, the distribution of microsaccade direction reflected the perceived motion direction. When mimicking the visual consequences of blinks and microsaccades, by introducing a transient visual interruption (blank) or a small shift of the stimulus (microshift), we found that a blank significantly facilitated a switch in percept, however, only toward coherent motion. Interestingly, a specific interaction between blinks, blanks, and microshift was found with respect to the microsaccade rate. While all events led to a significant reduction in microsaccade rate, this reduction started notably before the onset of a blink. Additionally, a subsequent increase in microsaccade rate above the baseline was found only for the blank.

### Overall Rate Changes

Independent of the fact that the length of the percept was significantly longer for coherent compared with component motion, we found a significantly higher overall rate of blinks and microsaccades for coherent motion. Our results further indicate that this difference might be explained by the modulation over time with respect to the switch event, as discussed below.

### Temporal Modulation of Blinks Dependent on the Perceptual Event

With regard to the time-resolved modulation in blinks, the manual response indicating a switch was either preceded by a decrease or followed by an increase in eye blink rates depending on the type of percept subjects switched to. Previous studies have reported this modulation (Ito et al., 2003; van Dam and van Ee, 2005, 2006) and have also found a percept-specific influence of blinks (Nakatani et al., 2011; Otero-Millan et al., 2012).

#### Increase of Blink Rate After the Indicated Perceptual Switch

An increase in blink rate occurred exclusively after the indicated switch to the coherent motion. The difference between the two percepts excludes several possible causes for the modulation of blink rate. Ito et al. (2003) argue that blink modulation reflects response preparation. This has been further supported by van Dam and van Ee (2005) who found that blinks increase not just for the perceptual switch report but also for random button presses. Similar results with regard to manual key presses, though in a different paradigm, were found by Cong et al. (2010) who showed that blinks are entrained by rhythmic finger tapping. The differences between the modulation of blinks around the two percepts in our study reveal that it is not a mere representation of the motor response and its preparation.

Another possible cause for the increase in blink rate is related to attentional processes. Many studies have found that at the end of an attentional period or at the end of task-relevant information, there is an enhancement of blinking (Wascher et al., 2015). The reporting of the switch in our study could be considered the end of an attentional period or a task-relevant perceptual event. In other words, to relate to the question of cause or consequence, it seems that blinks are a consequence of the coherent switch. However, this does not explain why the influence is specific to the coherent percept. It is, therefore, an interesting consideration that this difference in blinking reflects a difference in the internal process that underlies the two percepts. The two interpretations are not only quantitatively different (one motion direction vs. two) but might also be qualitatively different in the sense that only the component motion might include an additional calculation of depth.

Another possible explanation could be related to the difference in perceptual dominance, which could bias attention toward one of the two precepts. However, previous findings do not support an interpretation based on perceptual dominance. Neither the described idea that blinks lead to the preferred percept (Nakatani et al., 2011), nor the finding that a “surprise” stimuli (which would correspond to the less likely non-preferred percept) causing a reduction in blinks (Bonneh et al., 2016) is consistent with our data. Nevertheless, the deviation in blink behavior as revealed by our study suggests a difference in the internal process associated with the two perceptual switches. Many studies have shown that perceptual changes, based on a change in the sensory input, is associated with an increase in blink rate (Ohdra, 1995; Siegle et al., 2008; Oh et al., 2012; Bonneh et al., 2016; Hoppe et al., 2018). Such increase is further modulated by internal factors such as the interpretation of the sensory input as target or neutral stimulus (Brych and Händel, 2020). It is therefore an interesting observation that the usually observed increase in blink rate is not present after a perceptual switch to component motion. It suggests a difference in the underlying mechanism or internal consequence of the two perceptual switches. While the subject is aware of the perceptual switch (and reports it), it seems not aware that the switch to coherent is resembling a real switch based on changing sensory input, whereas the switch to component motion does not. Therefore, blink behavior is likely a marker to detect differences in percept-related processes that are not necessarily evident from the subjective experience.

It is important to note that the exclusivity of the blink modulation for one specific percept was not due to differences in the physical direction of the perceived motion direction. We addressed this by changing the direction of the stimulus. This was done to understand (1) if the vertical movement of the eyes during a blink (Collewijn et al., 1985) are linked to the perceived motion and (2) if the direction of the two motion percepts matters. It is known that the visual system is biased toward the cardinal directions, on a neuronal level and a perceptual one (Schluppeck and Engel, 2010; Girshick et al., 2011). If the coherent motion is following a cardinal direction while the component one is not, this could cause the system to treat perceptual interpretations differently. However, we found that the direction of the perceived motion had no significant effect on the blinking pattern. Hence, the difference between the percepts is, most likely, not due to any preference of physical directions, but rather due to some internal process.

#### Decrease of Blink Rate Before the Indicated Perceptual Switch

Our second main finding was a decrease in blink rate before the response indicating a switch. It is hard to tell if this decrease was percept specific, since it did not reach significance before the reported switch to component percept in the second experiment, although with higher power. This could indicate a weaker or less stable effect compared with the switch to coherent motion.

In general, both perceptual changes are internal events in our task, which needed to be reported and, therefore, should have drawn attention toward them. Studies have reported that people tend to suppress their blinks during moments of increased attention (Hoppe et al., 2018) and even before the onset of a task-relevant stimulus (Veltman and Gaillard, 1998; Hoppe et al., 2018). This suppression occurs even for stimuli outside the visual modality (Bauer et al., 1985), indicating the involvement of a more general, vision-independent attentional mechanism. The allocation of attentional resources caused by the switch in percept might have introduced the decrease in blink rate. This interpretation, however, would mean that the decrease happened as a consequence of the perceptual switch.

Another possibility is that the reduction in blinking is not only a result of the perceptual switch but also a likely cause. Indeed, increased fixation duration has been shown to lead to perceptual switching in other studies (Ellis and Stark, 1978; Nakatani and van Leeuwen, 2005), and since blinking interrupts fixation, the suppression of blinks might facilitate a perceptual switch. Unfortunately, it is difficult to conclude with certainty as to which event, the switch or the reduced blinking, occurred first, simply because there was no objective measure of the internal perceptual switch itself. However, we have a strong indication as to when the perceptual switch happened by looking at the blank results (Figure 3). Here, it is clear that the blank must have introduced the switch and not the other way around. This allowed us to mark the period in which the perceptual switch likely occurred, namely, between blank and response. Figure 2 clearly shows that the time of blink reduction before the perceptual switch overlaps with the time when the switch-introducing blank occurred. This indicates that the reduction in blink probability was not a result of the perceptual switch but temporally co-occurring. Interestingly, multisecond interruptions in ambiguous stimuli have been shown to stabilize percept (Leopold et al., 2002; Noest et al., 2007).

In summary, although one of our initial goals was to see if blinks act as a cause for perceptual switches due to their retinal consequences, we found that their role is different. Indeed, it is not the blink occurrence and the corresponding visual interruption that facilitates a switch, but rather the absence of a blink that does.

### Modulation of Microsaccades (Rate and Direction)

In experiment 2, we looked at the role of microsaccades in the ambiguous plaid stimulus and controlled for their retinal consequence by adding microshifts to the stimulus. We found that the overall microsaccade rate was higher for the coherent than the component percept. Additionally, we found a reduction in microsaccade rates specifically before the switch to the component percept. Please note that the discrepancy to other studies, reporting that an increase in microsaccades can introduce a perceptual switch, is most likely due to the different ambiguous stimuli used (Troncoso et al., 2008; Otero-Millan et al., 2012). These studies used the rotating snakes and the Enigma illusion, both of which alternate between movement and stationary percepts. Hence, it is likely that microsaccades specifically facilitate a switch to a motion percept. The plaid stimulus, used in our experiments, does not have switches between movement and no movement percept, but involves switching between different types of motion. Using a more comparable ambiguous apparent motion stimulus, a reduction before a perceptual switch has been reported before (Laubrock et al., 2005, 2008). These authors further argued that the microsaccade modulation might possibly precede the internal switch, indicating a possible causal role of microsaccades. As discussed above, we believe that our observed blank-introduced perceptual switch is a strong indication as to when the perceptual switch happened with respect to the response, namely, between −900 and −500 ms before the response (Figure 3). The timing of reduction in microsaccade rate as shown in Figure 5 (between −300 and 0 ms), therefore, suggests that the effect happened between the perceptual switch and the response, given an average reaction time of about 500 to 700 ms to an actual external stimulus change (van Dam and van Ee, 2005, 2006; Laubrock et al., 2008; Baker and Graf, 2010). Although it is not possible to tell with absolute certainty if the decrease in microsaccade rate follows the internal switch, the decrease only before the response indicating a switch to component percept argues against a mere consequence of response preparation.

Interestingly, we found that the direction of microsaccades is linked to the direction of the ongoing percept. Specifically, as shown in Figure 5, we found that while the overall direction was mainly opposite to the coherent motion, this proportion was reduced during component percept, and at the same time, the proportion of microsaccades in the direction opposite to the two possible component motion directions was increased. Since the direction is opposite to the percept, it is likely that the percept draws the eyes in the direction of perceived motion, and the detected microsaccade is a saccade back to the required position of fixation. This could mean that the microsaccades we observe are some sort of small optokinetic nystagmus (OKN), which is a well-known phenomenon that is triggered by moving background stimuli introducing optic flow. It consists of a slow phase in the direction of the optic flow and a short, fast jump back toward the center of the visual field. OKN is greatly reduced if visual fixation is demanded (Murphy et al., 1975). However, even during fixation of a stationary target, small eye movements, affected by a moving background, can be observed. For instance, Re et al. (2019) found that microsaccade directions are influenced by, and correspond to, the direction of moving dot clouds that are attended during fixation. While Laubrock calls them “OKN-like rudiments” (Laubrock et al., 2008), Pola and colleagues note that these residual movements have a rather complex relationship with the OKN (Pola et al., 1995). Further studies will need to clarify if the direction of microsaccades are a consequence of the percept or lead to the specific perceptual interpretation. What, however, is clear from our results is that, the microsaccade direction and the perceptual interpretation of sensory input are not independent of each other.

### The Effect of External Events: Blanks and Microshifts

Blanks and microshifts are external events that were initially planned as controls for the visual consequences of blinks and microsaccades. Interestingly, they have a different effect on perceptual bistability compared with their corresponding eye movements. One main finding was that the blanks introduced a switch to the coherent motion in experiment 2. Please note that the effect is also visible in experiment 1 (Figure 3), but the lower power in experiment 1 might have prevented significance. Two questions arise through the finding: (1) Why do blanks, but not blinks, introduce a switch despite their similar visual consequences and (2) Why do blanks specifically introduce a switch to the coherent percept?

With regard to the first question, one should bear in mind that even though blinks and blanks have a similar consequence on the retinal image, they are intrinsically different (Deubel et al., 2004; Higgins et al., 2009; Golan et al., 2018). Deubel et al. (2004) found that adding a blank after a saccade can counteract the reduced detection of target displacement due to saccadic suppression, but a blink after a saccade does not have the same effect. A similar finding was also reported for blink suppression, wherein introducing a blank period after a blink reduces the displacement suppression. The idea is that an external interruption due to a blank introduces a need to recompute the post-saccadic target location, whereas if the interruption is due to a blink, no such need is generated (Higgins et al., 2009). In other words, interruptions or small changes during blinks are generally ignored (Maus et al., 2017). This means that the oculomotor system treats an internal event such as a blink, differently from a blank. A difference between the two is also found on a neural level. A higher activity in several visual areas have been reported for blanks, but not blinks (Gawne and Martin, 2000, 2002; Golan et al., 2018), and blinks (both voluntary and spontaneous) along with self-initiated blanks are associated with a decrease in activity in higher visual areas, whereas unpredictable external darkening causes an increase in higher-level areas (Golan et al., 2018). A difference in perceptual consequence following a blink vs. a blank is, therefore, not surprising. Moreover, we must note that, in our experiments, there is also an additional difference between the two, namely, that the blank causes interruptions in the stimulus and not the entire visual scene like that of a blink.

However, the specificity of the perceptual change due to a blank is somewhat surprising. Blanks often led to a switch to coherent percept. This was the preferred interpretation of the stimulus. This could indicate that if a certain interpretation of a sensory input is preferred, anything that causes one to reassess/recompute the input will tend to switch the perceptual interpretation to the preferred one. Once we reach this preferred perceptual interpretation, we are more likely to blink, assuming that all relevant information has been assessed. It is important to point out that our findings might be specific for the ambiguous plaid stimulus where the two percepts are clearly based on different internal processes. While the component percept interprets the stripes separately due to different depths, the coherent percept is based on an integration over the two stripe stimuli. The investigation of other bistable stimuli can clarify this specificity.

With regard to microshifts, we did not find a significant modulation. This suggests that any possible effect of microsaccades is not due to the visual perturbation they introduce.

### Modulation of Microsaccade Rate Around Internal and External Events

We found that the microsaccade rate, although mostly constant around perceptual switches, was modulated around blanks, microshifts, and blinks, but with a difference in temporal dynamics around the internal (blinks) vs. the external (microshifts and blanks) events. Specifically, though there was a continued microsaccade reduction for ~250 ms after the event offset, this decrease started only after the onset of external events, but clearly before the onset of the internal event.

With regard to the blanks, the modulatory pattern introduced by the blank followed the typical microsaccade rate signature, characterized by a decrease, followed by an increase and a return to baseline (Bonneh et al., 2016). This modulation has been observed during other tasks and was suggested to be the reaction to sudden changes in visual input, such as display changes as well as to internal attention capturing processes (Engbert and Kliegl, 2003; Betta and Turatto, 2006; Pastukhov et al., 2013; Gao et al., 2015). Our blanks interrupted the visual information intake likely leading to a reassessment of visual input, which required the allocation of attention. However, microshifts and blinks did not show an increase in microsaccade rate after the decrease. A possible explanation stems from the fact that during the shift, there still is visual input, whereas during the blank, there is no visual information at all, which might generate a stronger need for reevaluation. This again would not happen after a blink, since a blink is self-introduced and provides no reason to assume that the input has changed. With regard to the internal blink event, we found that the actual decrease started earlier than for the external events, namely, around 200 ms before blink onset. It has been shown that microsaccades are suppressed when there is an expected visual stimulus followed by a response (Betta and Turatto, 2006). An expected change in sensory input due to a blink could trigger the same mechanism. However, it must be noted that the suppression reported by Betta and Turatto (2006) was specific to sensory information that should trigger a motor response and, therefore, be linked to response preparation, as argued by the authors. The predicted sensory change caused by a blink is not task relevant and is, as mentioned earlier, ignored by the system. That no microsaccades were detected during a blink is a result of our video-based eye tracker, where it is not possible to detect microsaccades when the eyes are closed. We conclude that, although the reason for the suppression of microsaccades before blink onset is not certain, our findings clearly indicate an interaction between blinks and microsaccades.

## Summary and Conclusions

Our study on blinks and microsaccades during a visual bistable task indicates that the execution of these eye-related movements is related to internal perceptual processes and that these movements influence each other's probability. The fact that different perceptual interpretations of the same sensory input are accompanied by a different eye movement pattern further suggests a difference in the internal process associated with the two perceptual switches. Such a difference is not evident from the subjective perceptual experience. The analysis of eye movements can therefore differentiate between distinct cognitive processes that might otherwise go undetected. Additionally, our findings suggest that eye movements might play a role in stabilizing percept.

## Data Availability Statement

The raw data supporting the conclusions of this article will be made available by the authors, without undue reservation.

## Ethics Statement

The studies involving human participants were reviewed and approved by Ethic commission of the Institute of Psychology of the Philosophical Faculty II of the Julius-Maximilians University Würzburg. The patients/participants provided their written informed consent to participate in this study.

## Author Contributions

MB was involved in the development of the research design, data collection, formal analysis and the writing of the results and methodology. SM was also involved in the development of the research design, data collection of experiment 1, initial analysis and the writing of the introduction and discussion. The project was overseen and supervised by BH. All authors contributed to the article and approved the submitted version.

## Conflict of Interest

The authors declare that the research was conducted in the absence of any commercial or financial relationships that could be construed as a potential conflict of interest.
