# Supplementary material for: The Role of Blinks, Microsaccades and their Retinal Consequences in Bistable Motion Perception
Source: Front Psychol. 2021 Apr 8;12:647256. doi: 10.3389/fpsyg.2021.647256 (PMC8061730; doi:10.3389/fpsyg.2021.647256)
Supplement: Supplementary file 1 [file Data_Sheet_1.docx]

**Supplementary Material**

**The Role of Blinks, Microsaccades and their Retinal Consequences in Bistable Motion Perception**

Mareike Brych1*, Supriya Murali1*, Barbara Händel1

1 Department of Psychology III, University of Würzburg, Würzburg, Germany

*these authors have contributed equally to this work and share first authorship

**Details on microsaccade detection**

We detected saccades based on an algorithm described by Engbert and Kliegl (2003). We further excluded all saccades which had a logarithmic peak velocity more than three absolute deviations away from the median (Leys et al., 2013). As an example, Fig. S1A shows the peak velocity plotted against the amplitude of all detected saccades in one trial of one participant. The excluded saccades based on peak velocity are marked by yellow stars. We further marked those events detected as saccades, which happened within 20 ms of a blink (Fig. S1A, red crosses). These events show a much larger amplitude with higher peak velocity compared to other saccades. Additionally, they primarily show a downward direction when happening before the blink and an upward direction when happening afterwards (Fig. S1B). This suggests, that these detected events are of different nature than saccades during undisturbed gaze and rather blink-induced. Importantly, our analysis regarding saccadic eye movements was confined to microsaccades, which were defined by a maximal amplitude of 1°. Only 0.2% of microsaccades were excluded by the velocity criterion and additional 0.01% were excluded around blinks (Fig. S1C).

**A B C**


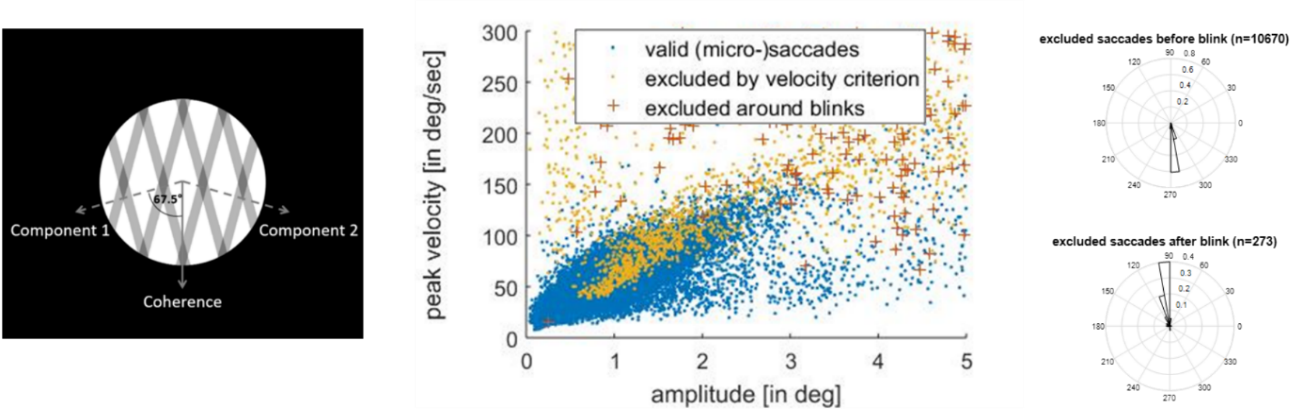

*Figure S1.* A. Example of saccade detection in one trial of one participant. Yellow dots mark saccades, which were marked based on the velocity criterion. Red crosses mark saccades, which were detected ± 20 ms around a blink. Blue dots represent valid saccades. B. Direction of detected saccades 20ms before or after a blink of all participants. C. Microsaccade detection of all trials and all participants.

**Additional blink results**

In addition to the analysis of the normalized blink/blank rate around perceptual switches averaged over stimulus rotations, we present the data for the four different stimulus rotations separately for experiment 1 (Fig. S2) and 2 (Fig. S3).

Following the significant effect of blink increase after the response indicating a perceptual switch to coherent motion, there is a clear increase for all four rotations. Similarly, all rotations show a decrease before the perceptual switch, however, as expected from the averaged results, this decrease is more pronounced for switches to component percept. We therefore assume that blink related effects are independent of the movement direction.

The effect of blanks on the perceptual switch is prevalent for switches to coherent motion only, as expected from the analysis of the averaged data, but the effect seems to be mostly confined to downward motion of one component in experiment 1 (see second subplot of figure S2 D) . However, experiment 2 again shows a motion direction independent effect. Please note that rotations 22.5° and 90° (two right most rotations in figure S3) were chosen because of the prevalence of microsaccades in the horizontal direction and therefore could include microshifts, but not blanks.

A

B

C

D

E

*Figure S2*. Normalized blink/blank rate separated for the four different stimulus rotations in *experiment 1*. A. Stimulus illustration for the rotations 0°, 67.5°, 112.5° and 180° (from left to right). Solid line represents direction of coherent motion, dashed lines represent direction of component motion. B. Normalized *blink* rate for the four rotations separately when a switch to coherent motion occurred. The mean is presented in the main manuscript in figure 3A. C. Normalized *blink* rate for the four rotations separately when a switch to component motion occurred. The mean is presented in the main manuscript in figure 3B. D. Normalized *blank* rate for the four rotations separately when a switch to coherent motion occurred. The mean is presented in the main manuscript in figure 4A. E. Normalized *blank* rate for the four rotations separately when a switch to component motion occurred. The mean is presented in the main manuscript in figure 4B.

A

B

C

D

E

*Figure S3*. Normalized blink/blank rate separated for the four different stimulus rotations in *experiment 1*. A. (From left to right) Stimulus illustration for the rotations 0°, 67.5°(blank trials), 22.5° and 90° (microshift trials). Solid line represents direction of coherent motion, dashed lines represent direction of component motion. B. Normalized *blink* rate for the four rotations separately when a switch to coherent motion occurred. The mean is presented in the main manuscript in figure 3C. C. Normalized *blink* rate for the four rotations separately when a switch to component motion occurred. The mean is presented in the main manuscript in figure 3D. D. Normalized *blank* rate for the two rotations separately when a switch to coherent motion occurred. The mean is presented in the main manuscript in figure 4C. E. Normalized *blank* rate for the two rotations separately when a switch to component motion occurred. The mean is presented in the main manuscript in figure 4D.

**References**

Engbert, R., & Kliegl, R. (2003). Microsaccades uncover the orientation of covert attention. *Vision Research* 1035–1045.

Leys, C., Ley, C., Klein, O., Bernard, P., & Licata, L. (2013). Detecting outliers: Do not use standard deviation around the mean, use absolute deviation around the median. *Journal of Experimental Social Psychology*, 764-766.
